# ACTonFOOD: opportunities of ACT to address food addiction

**DOI:** 10.3389/fpsyg.2015.00396

**Published:** 2015-04-09

**Authors:** Roberto Cattivelli, Giada Pietrabissa, Martina Ceccarini, Chiara A. M. Spatola, Valentina Villa, Annalisa Caretti, Arianna Gatti, Gian Mauro Manzoni, Gianluca Castelnuovo

**Affiliations:** ^1^Psychology Research Laboratory, Istituto Auxologico Italiano IRCCSVerbania, Italy; ^2^Department of Psychology, Catholic University of MilanMilan, Italy; ^3^Department of Psychology, University of BergamoBergamo, Italy; ^4^Private PracticeParma, Italy

**Keywords:** Acceptance and commitment therapy (ACT), food addiction, obesity, eating disorders, binge eating disorder, bed, weight management, weight loss, CBT, clinical psychology, health psychology, psychotherapy

Being overweight is a growing problem worldwide, and is becoming an epidemic both in Europe and the United States. Recent reports show that 64% of U.S. adults are overweight, and this a rate continues to rise (Lifshitz and Lifshitz, 2014). In the United States, the economic burden on the healthcare system related to this issue is approximately 100 billion dollars (Cawley et al., 2014; Specchia et al., 2015). The economic burden Europe is similar to that in the United States (Pietrabissa et al., 2012; Lehnert et al., 2014).

Health risks frequently related to being overweight include psychological difficulties, such as depression and stigma, and physical impairments, such as cardiovascular, oncological, metabolic, or osteoarticular diseases (Deitel, 2002; Forman and Bulwer, 2006; Castelnuovo et al., 2014; Knäuper et al., 2014). The main challenge in facing obesity and the associated acute or chronic diseases is promoting the development and implementation of comprehensive weight-management programs, which often include a combination of physical activity, diet, and psychological intervention (Kramer et al., 2011, 2014). Nevertheless, the effects of these programs are generally not long-lasting (Castelnuovo and Simpson, 2011). According to recent findings, the maintenance of achieved weight-loss lasts only for a short period of time (Gifford and Lillis, 2009; Cooper et al., 2010; Knäuper et al., 2014).

In general, availability, costs, treatment adherence, and long-term efficacy are important limitations of these varieties of approaches (Byrne et al., 2003; Manzoni et al., 2009; Cesa et al., 2013; Castelnuovo et al., 2014). Frequently, obese patients regain about 30% of the weight lost during treatment within 1 year and they typically return to their baseline weight within 3–4 years (Castelnuovo et al., 2011). Traditional behavioral and cognitive-behavioral treatments included in multi-disciplinary interventions, rarely used as stand-alone programs, are often considered a gold standard to face “Globesity” (Lifshitz and Lifshitz, 2014), which refers to the global emergency of overweight individuals (Deitel, 2002; Avena et al., 2012b; Pietrabissa et al., 2012; Castelnuovo et al., 2014). However, long-term results are generally poor (Cooper et al., 2010).

CBT-based programs show good outcomes for a majority of the obese population, as they promote control strategies, such as restrictive dietary intake, physical activity prescriptions, and thought suppression or cognitive restructuring (Forman et al., 2007, 2013; Cooper et al., 2010). However, according to the research, outcomes of these programs usually do not last long (Foreyt and Poston, 1998; Byrne et al., 2004; Cooper et al., 2010). Emerging models to assess obesity now point to the core role of food addiction (FA) to conceptualize obesity not only as an effect of an unhealthy lifestyle but also as an effect of the role of underlying psychological factors (Riva et al., 2006; Gearhardt and Corbin, 2011; Gearhardt et al., 2011a,b; Avena et al., 2012a; Boggiano et al., 2014; García-García et al., 2014).

According to these models, excessive food consumption is similar to substance addiction (Gearhardt et al., 2012). Addictive behaviors occur in various forms, including overeating (Shaffer et al., 2004). In some obese individuals, compulsive overeating symptoms mirror signs associated with other compulsive behaviors, such as those seen with addictions (James et al., 2004; Volkow and Wise, 2005; Volkow and O'Brien, 2007; Gearhardt et al., 2011a). Evidence suggests that a certain number of obese individuals without inherited metabolic vulnerabilities encounter significant weight-loss difficulties and signs of FA (Gearhardt et al., 2009, 2012; Davis et al., 2011).

While abstaining from substances and alcohol and establishing positive coping mechanisms is encouraged for those with addiction, it is impossible to abstain from feeding. Additionally, the consumption of some foods is related to physiological and psychological modifications that are generally associated with substance addiction, such as withdrawal, tolerance, loss of control, cravings, and impulsivity (Volkow and Wise, 2005). Palatable food can activate the brain reward system through fast input sensors and post-binging outcomes, which results in higher glucose levels in the brain and the blood (Garber and Lustig, 2011). The reward circuit activated by appetizing food can also be directly activated by psychotropic substances (Di Leone et al., 2012).

A majority of obese patients display high levels of “food cravings,” which are addiction-like symptoms toward food. These patients do not respond effectively to weight-loss interventions (Avena et al., 2011). This condition leads to an increasing desire to eat to control uncomfortable feelings and negative emotional states. The amount and type of food eaten and the way in which such unhealthy eating occurs varies from person to person (Hill et al., 2014).

Despite the absence of accurate data regarding the prevalence of FA in the obese population, interventions aimed to face both overweight and FA, including addiction-like treatment elements, could show better outcomes compared to standard weight-loss treatments (Avena et al., 2012a). According these preliminary, but promising findings, new frontiers in weight-loss treatments should consider the role of FA as a fundamental psychological factor underlying difficult weight management situations (Gearhardt and Brownell, 2013; Gearhardt et al., 2014; Hebebrand et al., 2014; Innamorati et al., 2015), and foster appropriate addictive-behavior interventions (Ceccarini et al., 2014).

Different lines of research have investigated elements linked to successful and unsuccessful weight management and have designed programs targeting these factors (Gifford and Lillis, 2009; Lillis et al., 2009; Barnes and Tantleff-Dunn, 2010b; Schuck et al., 2014). Individuals who regain previously lost weight present a narrow range of coping skills. In fact, these individuals tend to be avoidant, impulsive and, in many cases, eat emotionally (Avena et al., 2011; Schag et al., 2013). On the other hand, better outcomes are seen among people with higher flexibility, acceptance, and more commitment to health habits (Gifford and Lillis, 2009).

In their seminal work, Lillis et al. (2009) suggested addressing treatments and resources that do not directly affect cravings or coping skills or that merely focus on weight management, but introduce an acceptance and mindful-based approach to treat obesity and overweight. Teaching and training skills to embrace emotional discomfort and difficult thoughts, to reduce experiential avoidance, and to foster persistence with a value-based and value-oriented behavior, should represent significant progress for long-term behavior modification in various fields (Lillis et al., 2011; Weineland et al., 2012).

Acceptance and commitment therapy, referred as ACT, is used widely to promote healthy lifestyles and psychological well-being in many contexts, including addictions, cardiovascular diseases, and eating disorders (Prevedini et al., 2011; Weineland et al., 2012; Spatola et al., 2014a,b; A-Tjak et al., 2015). For example ACT-based intervention was used with promising results to improve exercise tolerance in low-active women (Ivanova et al., 2014). The psychological flexibility model, grounded in functional contextualism and directly derived from the relational frame theory, which is a behavioral account of language and cognition, faces the challenge of the human condition to promote better adaptation to different life-contexts. The clinical application of this model is a technology, ACT, that is under continuous revision and is marked by a high level of flexibility, a range of clinical and sub-clinical applications, and a strong link to basic science (Gifford and Lillis, 2009; Barnes and Tantleff-Dunn, 2010a).

ACT, established in behavioral technologies and sciences, can integrate gold standard practices to improve compliance, foster behavior modification, and promote the continuous monitoring of target behaviors. Furthermore, functional, and not merely topographical, adaptations of ACT are needed to nurture behavioral change to account for the sociocultural diversity of different contexts and improve effectiveness of interventions for different contexts (Cattivelli et al., 2012a,b; Drossel et al., 2014). The focus of acceptance and mindfulness-based treatments is to increase flexibility, not through the replacement of dysfunctional thoughts or the introduction of powerful control strategies (e.g., cognitive reappraisal), but by teaching the patient to be present and consistent with freely chosen values (Barnes and Tantleff-Dunn, 2010b).

Teaching acceptance and mindfulness skills to handle difficult feelings and thoughts may be particularly helpful for those who are inflexible and who tend to avoid emotional distress (Lillis et al., 2009). ACT offers a range of valid applications for obesity and weight-management, from individual therapy to group settings, with both in- and outpatients. Further, ACT offers different ways to provide treatments, including phone consultation and web-based interventions, with highly efficient resource allocation, valuable outcomes, and efficiency. Recent findings show excellent results in this area (Bricker et al., 2013; Schuck et al., 2014). The opportunity to introduce ACT web-based protocols to target obesity is probably a valid innovation in the range of treatments for overweight regarding cost-resources efficiency. Recent literature on smoke cessation using an ACT approach has yielded important outcomes and innovations in content delivery (Schuck et al., 2011). The adaptation of content to be freely shareable and flexible in the maintenance phase after a specific program or stand-alone treatment could be a significant innovation in weight control science and could reach different populations to increase the social influence of acceptance-based programs in health promotion.

Changing the focus from topography to function, without directly aiming focusing on psychological distress but addressing the disposition to control or avoid difficult emotions and thoughts, is the key feature of ACT. ACT may be relevant to treating overweight and obesity because of the long-term weakness of more traditional approaches (Prevedini et al., 2011). This idea is consistent with the literature on addictions and substance abuse, which suggest that a fundamental way to maintain abstinence is to increase the individual's openness to psychological struggles or triggers; the pain literature shows similar findings (Gifford and Lillis, 2009; Lillis et al., 2011; García-García et al., 2014). Therefore, treatments for obese individuals with high FA levels should include teaching a greater tolerance of psychological distress, increasing the ability to engage in value-oriented actions, and reducing the struggle to control difficult emotions and thoughts and develop better management of emotional eating, thus fostering long-term behavioral change.

Forman et al. (2007), compared control strategies using an acceptance and mindfulness approach, and found that, in presence of higher levels of food cravings, participants obtained better outcomes in the ACT-consistent condition. These preliminary findings support the introduction of acceptance and mindfulness-based interventions in the context of traditional multi-disciplinary interventions for obesity, especially when targeting non-respondent and highly-avoidant individuals (Forman et al., 2007). An explicit inclusion of FA and experiential avoidance measures, in particular for non-responders to standard treatments, could represent a first step to tailored interventions for individuals presenting high levels of avoidant and addiction-like behaviors.

Thus, the inclusion of ACT in well-established multi-disciplinary intervention to replace or use in combination with CBT may foster behavioral changes that are consistent with health habits, particularly for highly avoidant patients (Lillis et al., 2011; Forman et al., 2013; Hawkes et al., 2014). The added value of acceptance and mindfulness-based treatments is not a short-term change; rather, it yields long lasting outcomes. Recent papers deploy in this direction, showing similar effects to traditional CBT at the end of treatment and better long-term outcomes at follow-up (Weineland et al., 2012; Forman et al., 2013). The identification of psychological factors, in particular FA, may help select individuals who require an intervention aimed to reduce experiential avoidance and promote value-base acting, thus, allowing an increase in the effectiveness and efficiency of existing treatments in combination with ACT. There is no clear consensus on the existence of well-defined criteria neither for FA nor, as pointed out by recent literature (Hebebrand et al., 2014), for Eating Addiction. Nonetheless, DSM5 seems to open to broader definitions of addictive behaviors, including non-substance related disorders (Hone-Blanchet and Fecteau, 2014; Meule and Gearhardt, 2014; Potenza, 2014). Thus, the debate is still open, as recent guidelines from Australian PA (Hay et al., 2014) point-out the need to provide more evidence supporting the use of ACT, or other growing-evidence treatments, for addictive-like eating. Despite this, promising results in the field of obesity associated with addiction-like behaviors toward food (Forman et al., 2013) suggest to develop further researches with ACT for obese non-responders, experiencing high levels of avoidance craving for palatable food. Hopefully, in the near future research will identify key elements of addiction recurring in the field of addictive eating and food dysfunctional consumption, and design interventions more tailored to face them.

## Conflict of interest statement

The authors declare that the research was conducted in the absence of any commercial or financial relationships that could be construed as a potential conflict of interest.
